# A new method for measuring treadmill belt velocity fluctuations: effects of treadmill type, body mass and locomotion speed

**DOI:** 10.1038/s41598-021-81951-9

**Published:** 2021-01-26

**Authors:** Steffen Willwacher, Kai Daniel Oberländer, Patrick Mai, Daniela Mählich, Markus Kurz, Till Koopmann, Dominik Fohrmann, Artur Kantarev, Uwe Gustav Kersting

**Affiliations:** 1 Department of Mechanical and Process Engineering, Offenburg University of Applied Sciences, Badstraße 24, 77652 Offenburg, Germany; 2 Institute of Biomechanics and Orthopaedics, German Sport University Cologne, Cologne, Germany; 3 School of Human Movement and Nutrition Sciences, The University of Queensland, St Lucia, QLD Australia; 4 Motesque Inc., Cologne, Germany; 5 Hochschule Fresenius, Cologne, Germany; 6 Department of Quality Technology & Mechanical Engineering, Mid Sweden University, Östersund, Sweden; 7 Institute of Sport Science, The University of Oldenburg, Oldenburg, Germany; 8 Institute of Exercise Training and Sport Informatics, German Sport University Cologne, Cologne, Germany

**Keywords:** Physiology, Bone quality and biomechanics, Biomedical engineering

## Abstract

Treadmills are essential to the study of human and animal locomotion as well as for applied diagnostics in both sports and medicine. The quantification of relevant biomechanical and physiological variables requires a precise regulation of treadmill belt velocity (TBV). Here, we present a novel method for time-efficient tracking of TBV using standard 3D motion capture technology. Further, we analyzed TBV fluctuations of four different treadmills as seven participants walked and ran at target speeds ranging from 1.0 to 4.5 m/s. Using the novel method, we show that TBV regulation differs between treadmill types, and that certain features of TBV regulation are affected by the subjects’ body mass and their locomotion speed. With higher body mass, the TBV reductions in the braking phase of stance became higher, even though this relationship differed between locomotion speeds and treadmill type (significant body mass × speed × treadmill type interaction). Average belt speeds varied between about 98 and 103% of the target speed. For three of the four treadmills, TBV reduction during the stance phase of running was more intense (> 5% target speed) and occurred earlier (before 50% of stance phase) unlike the typical overground center of mass velocity patterns reported in the literature. Overall, the results of this study emphasize the importance of monitoring TBV during locomotor research and applied diagnostics. We provide a novel method that is freely accessible on Matlab’s file exchange server (“getBeltVelocity.m”) allowing TBV tracking to become standard practice in locomotion research.

## Introduction

Treadmills are valuable tools for research on both human and animal locomotion as well as applied sport and medical diagnostics. They enable the study of human and animal motion under controlled conditions while requiring little laboratory space. Treadmills differ greatly in size, geometry, materials and actuation/control systems.

When locomoting on treadmills, the total body center of mass of humans and animals remains almost stationary in the horizontal direction relative to a global reference. However, horizontal velocity fluctuates relative to the moving reference frame of the treadmill belt. Treadmill belt velocity (TBV) fluctuation involves a complex interplay of friction forces between the belt and supporting surface, the actuation of the belt, and the control algorithms in powered treadmills^1^. Some more traditional powered treadmills rely on the inertia of the drive train (flywheel, heavy rollers etc.) to minimize TBV fluctuations. While there is evidence suggesting TBV fluctuation differs between different types of treadmills^1^, there has not been a systematic quantification of TBV fluctuation across different treadmills.

In dry conditions, the force of friction (F_fr_) is less than or equal to the product of the normal force (F_n_) and the coefficient of friction (µ)

1 $${\text{F}}_{{{\text{fr}}}} \le \, \upmu \, \cdot {\text{ F}}_{{\text{n}}}$$

While µ depends upon the properties of the interacting materials, F_n_ in human and animal locomotion is proportional to body mass and locomotion speed^2–4^. Therefore, TBV regulation likely differs between people of different body mass or when moving at different locomotion speeds. However, a systematic analysis of the effects of body mass and locomotion speed on TBV regulation in different treadmill types has not been published.

Quantifying TBV fluctuations is important because accurate values of average and instantaneous TBV are essential in the calculation of several fundamental physiological variables. For example, the calculation of the cost of transport (i.e., metabolic cost per distance^5^) or locomotion economy (i.e. metabolic energy consumption rate at a given locomotion speed^6^) for moving on a treadmill requires precise knowledge of the average belt velocity. Further, the quantification of external center of mass (CoM) power (i.e. cross product of ground reaction force and CoM velocity vectors) during locomotion necessitates precise tracking of instantaneous TBV^7,8^. In addition, while different approaches for the quantification of mechanical work during locomotion exist, most of them assume a constant TBV^9^. While there are precise measures to calculate average TBV (using, e.g., stopwatches or tachometers), the quantification of TBV fluctuations during the gait cycle is not commonly performed.

Further, when generalizing results from treadmill studies to field conditions, it should be considered whether the instantaneous CoM velocity pattern observed on a treadmill matches typical patterns for overground conditions. Nonetheless, TBV is not a standard variable reported in studies assessing locomotion related problems. Potentially, the lack of a time-efficient and feasible method to measure TBV fluctuation has contributed to the omission of this variable in the past.

Therefore, the goals of the present study were: (1) To develop a method for easily determining instantaneous TBV and TBV fluctuation using standard 3D motion capture system technology and (2) To analyze the effects of treadmill type, locomotion speed, and body mass on TBV regulation. We hypothesized that treadmill type, speed, and body mass would significantly alter TBV fluctuation.

## Materials and methods

### Participants

We recruited seven healthy, active, young participants (6 male; 1 female; body mass: 60.7–108.2 kg; body height: 1.63–1.98 m; age: 18–35) for this study. The participants gave written informed consent before data collections. The Ethical Commission of the German Sport University Cologne approved all methods employed in this study, which were in accordance with the Declaration of Helsinki.

### Experimental procedure

We collected 3D motion capture data while the participants were walking (1.0, 1.5, 2.0 m/s target speed) and running (2.5, 3.0, 3.5, 4.0, 4.5 m/s target speed) on four different treadmills: (1) a relatively high-cost, non-instrumented treadmill (Stellar med, h/p/cosmos sports & medical GmbH, Nussdorf-Traunstein, Germany), (2) a relatively low-cost, non-instrumented treadmill (F85, Sole, Stoke-on-Trent, UK), (3) an older, force-instrumented treadmill (#1), in which the treadmill belt and bed had been substantially worn out (Treadmetrix, Park City, UT, USA) and (4) a new force-instrumented treadmill (#2, Fully Instrumented Treadmill/FIT, Bertec, Columbus, OH, USA). Target speed was defined as the speed displayed on the treadmills’ control panels. Because these treadmills were located in different labs, we collected marker trajectory data on three different days while using different motion capture systems. We collected data for treadmills (1) and (2) using a Qualisys motion capture system (250 Hz, Miqus, Qualisys AB, Gothenburg, Sweden) on the same day; we captured data for treadmill (3) using a Vicon motion capture system (250 Hz, Bonita, Vicon Motion Systems, Oxford, UK), and data for treadmill (4) with an OptiTrack motion capture system (240 Hz, Prime17W, OptiTrack, Corvallis, OR, USA). All markers and marker patches were applied to the participants and the treadmills by the same operator. For each data collection, the user interface of the respective treadmill was set to the target speed and 17 s of data were collected for each participant at each speed.

**Figure 1:**
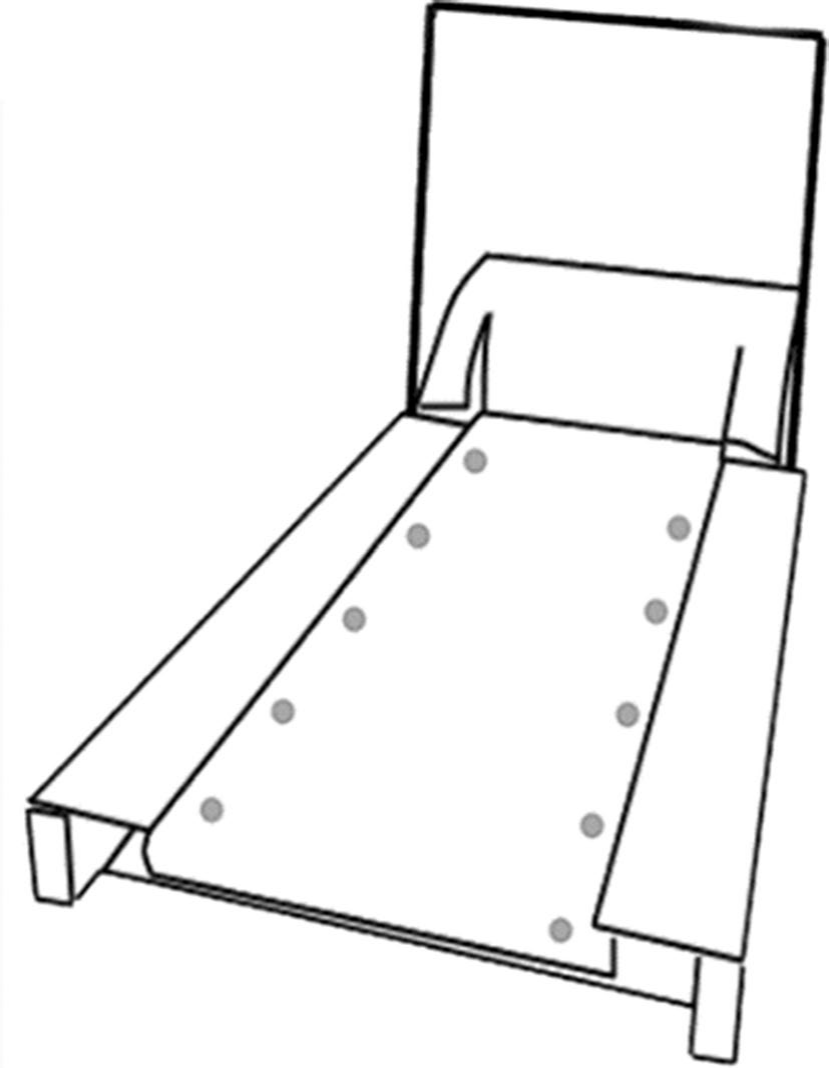
Schematic drawing of marker placement on the treadmills.

The operator attached circular cut-outs of retro-reflective adhesive foil (radius = 8 mm; 3 M, St. Paul, MN, USA) at regular intervals to the lateral aspects of the treadmill belts (Fig. 1). We chose the distance between cut-out markers such that ten of them (five per lateral side) were visible on the top side of the belt at each time. We used the tracked 3D coordinates of these markers to calculate TBV. For the application of the new method it is of critical importance that the treadmill is perfectly aligned with the antero-posterior axis of the laboratory coordinate system. We checked this assumption by checking the coordinates of two markers positioned in the front and back of the treadmill at the same medio-lateral position. This way, we could also verify that the treadmill inclination was zero during data collection.

Additionally, the operator attached seven markers to anatomical landmarks on the subjects’ pelvis and right leg: Right and left posterior superior iliac spine, right greater trochanter, right lateral femoral condyle, right lateral malleolus, right posterior heel, right tip of the great toe. Those markers were used for stance-phase detection.

### Data analysis

#### Gait event detection

We filtered the 3D coordinates of markers attached to the participants' pelvis and right leg using a 4th order, recursive, digital Butterworth low-pass filter (cut-off frequency: 6 Hz), implemented in Matlab (R2018b, The Mathworks, Natick, MA, USA).

For the walking trials, we extracted touchdown and toe-off events using a previously published method^10^ using the peak horizontal distances between markers placed on the pelvis and on the heel or toe to determine initial and final contact during the stance phase, respectively. For running trials, we applied a previously evaluated method which uses peak knee extension angles to determine initial and final ground contact^11^.

#### Belt velocity algorithm

**Figure 2:**
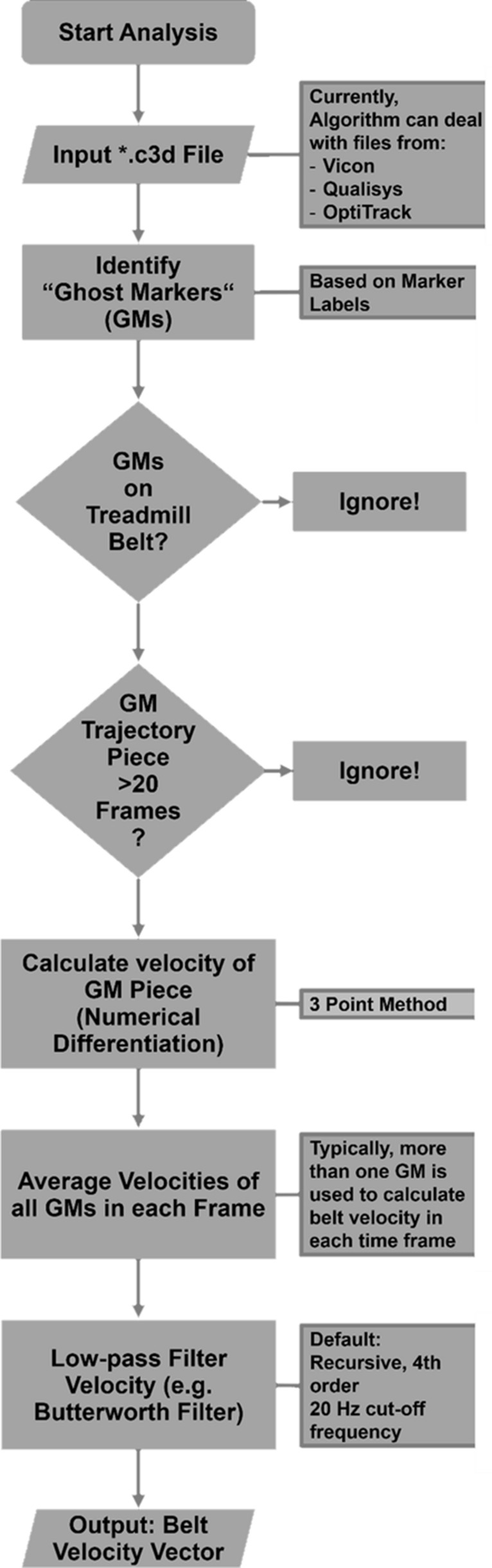
Description of the belt velocity algorithm.

Using the 3D coordinates of the reflective foil cut-outs attached to the treadmill belts, we developed an algorithm to determine the instantaneous TBV. The underlying code is freely available as a Matlab (The Mathworks, Natick, MA, USA) function through Matlab’s file exchange server (“getBeltVelocity.m”). Figure 2 shows a brief description of the individual steps of the algorithm.

The algorithm works with the coordinates of markers attached to the treadmill belt as “unlabeled marker” data to calculate TBV via time differentiation and by averaging over redundant markers. With this approach, there is no need for time-consuming labeling of the belt marker data. The algorithm reads marker data from “.c3d” files by using the Biomechanical Toolkit (BTK), an open-source framework for visualization and processing of biomechanical data^12^. The code separates the belt markers from other “unlabeled markers” or outliers (markers resulting, e.g., from reflections of apparel materials or short time flickering markers resulting from errors in the reconstruction procedure) by the following method: Initially, the user defines two volumes in the lab coordinate system in which the belt markers on the respective lateral belt sides are moving. The algorithm excludes all markers lying outside of those volumes from further analysis. Also, the algorithm excludes all markers that do not appear for at least 20 consecutive timeframes. Subsequently, the algorithm determines the horizontal belt velocity in the direction of movement for each belt marker, using the average marker distance over three frames and the known measuring frequency. The algorithm excludes outliers by checking if the calculated velocity is exceeding a user defined upper-limit of velocity. In this study, we used an upper limit of + 30% of the target speed as the upper-limit for outlier detection. Finally, the algorithm calculates the average from all markers’ velocities at each time frame and applies a 4th order, recursive Butterworth filter (default setting: 20 Hz cut-off frequency). The default settings were chosen to be similar to cut-off frequencies often used to filter marker data in the analysis of running mechanics^13–15^, but can be adapted for individual purposes.

We calculated the instantaneous TBV fluctuation using the novel method for every trial. Further, we subdivided each trial into individual gait cycles (from the touchdown of the right leg to the next ipsilateral touchdown) . We analyzed all gait cycles occurring in the 17 s data collection time, resulting in at least ten analyzed gait cycles for every trial. Afterwards, we extracted the following discrete parameters for further statistical analysis: Average TBV during stance phase, and during the entire gait cycle as well as the reduction of TBV during the braking phase of stance. These variables were normalized to the respective target speeds in order to exclude any trivial effects of target speed on these parameters during the statistical analysis. Besides TBV amplitude parameters, we also quantified the relative temporal occurrence of the minimum TBV during the stance phase.

### Statistical analysis

We extracted the parameters of interest for each gait cycle. We then averaged these values for each participant in each condition before implementing these averaged values into the statistical analysis. Descriptive statistics present the means ± one standard deviation of the sample data. To analyze the effects of treadmill types, locomotion speed and body mass on TBV regulation, we performed a two factor (treadmill type, locomotion speed) repeated measures analysis of variance (ANOVA) with body mass as a covariate using the ‘ranova’ and ‘fitrm’ functions of Matlab’s Statistics and Machine Learning Toolbox (R2018b, The Mathworks, Natick, MA, USA). All data underlying the statistical analyses are provided in Supplementary Material spreadsheet file.

## Results

**Figure 3:**
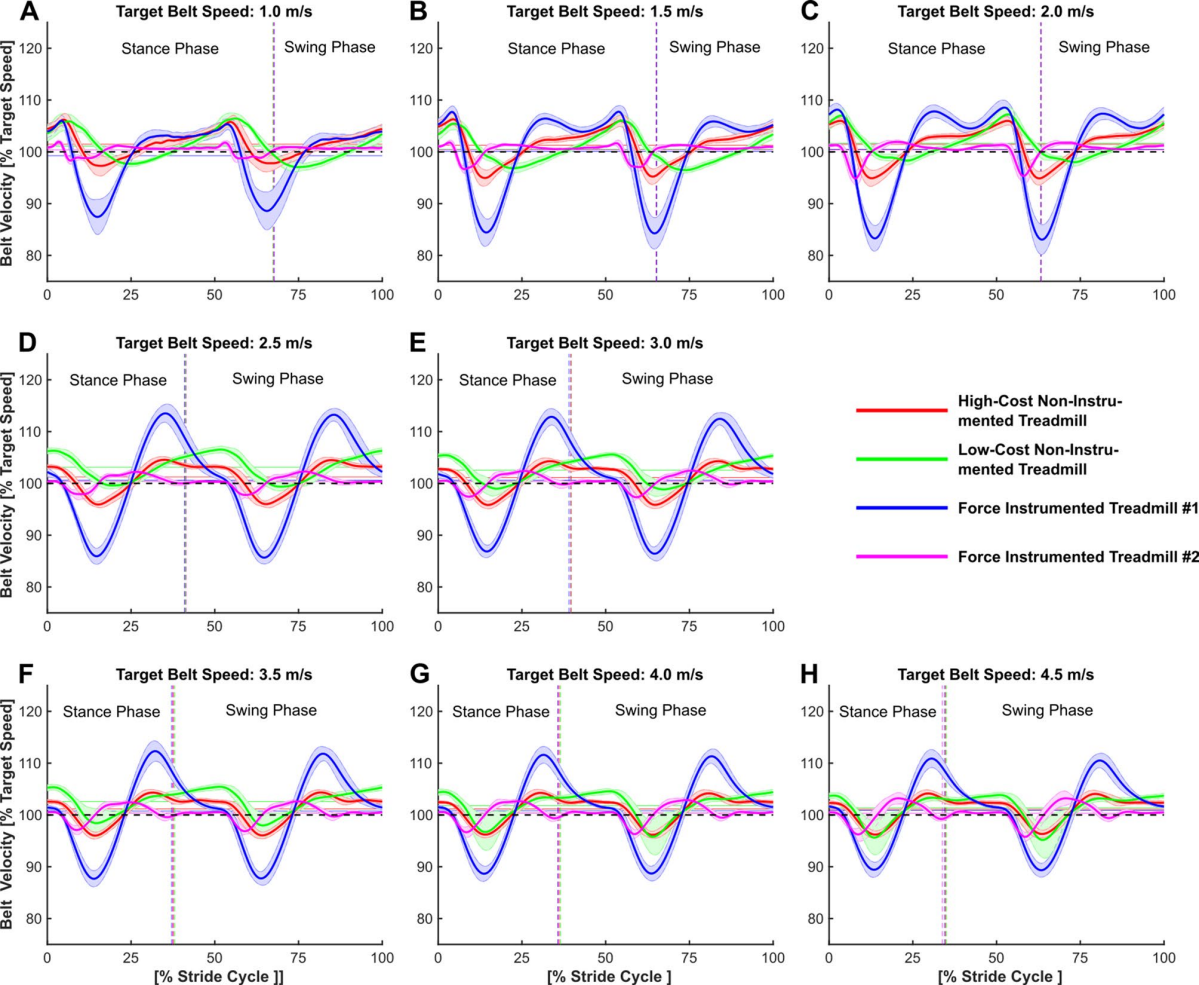
Ensemble averages of treadmill belt velocity profiles of the four different treadmill types analyzed at three different walking target speeds (1.0–2.0 m/s, **A**–**C**, respectively) and five different running target speeds (2.5–4.5 m/s, **D**–**H**, respectively). Solid lines indicate the mean (n = 7) of the particular treadmill condition. The shaded areas display ± one standard deviation around the mean. Data is time-normalized to the entire gait cycle (from right leg initial ground contact to next ipsilateral ground contact). Vertical broken lines indicate the final contact of the right foot with the ground (toe-off).

Treadmill type, locomotion speed, and body mass each significantly affected TBVs. Figure 3 illustrates the TBV behavior during the entire gait cycle between treadmills at all analyzed locomotion speeds and highlights the differences in amplitude and timing of TBVs.

**Figure 4:**
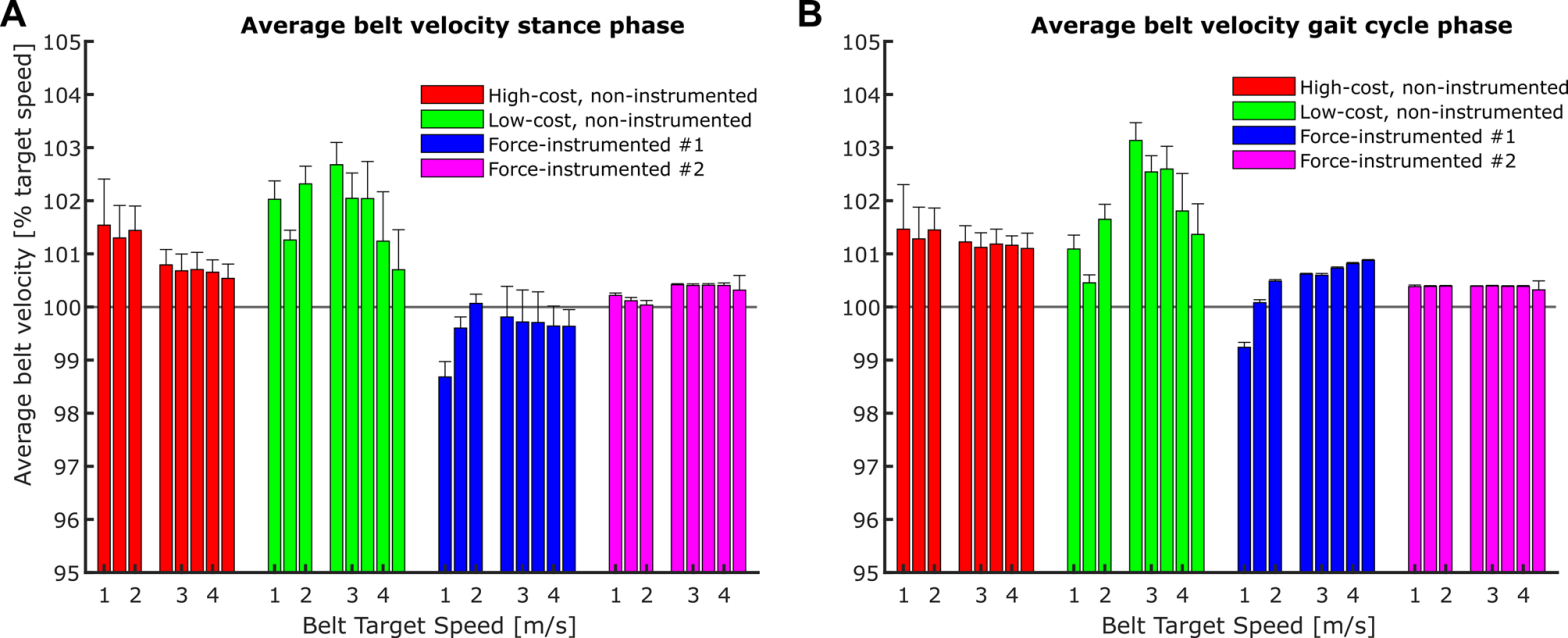
Average treadmill belt velocity (mean ± standard deviation) quantified for the stance phase (**A**) and the entire gait cycle (**B**) for four treadmills at different locomotion speeds. We separated the bars of the walking speeds (1.0 m/s to 2.0 m/s) from the bars of the running speeds (2.5 m/s to 4.5 m/s).

We observed significant main effects of treadmill type on the average TBV determined during the stance phase (p < 0.001, Fig. 4A) and the entire gait cycle (p < 0.001, Fig. 4B). When averaged over all subjects and locomotion speeds, the most pronounced deviations in average TBV were found for the low-cost, non-instrumented treadmill (during stance: + 1.8 ± 0.7%; during gait cycle: + 1.8 ± 0.9%), followed by the high-cost, non-instrumented treadmill (during stance: + 1.0 ± 0.4%; durig gait cycle: + 1.2 ± 0.1%), the force-instrumented treadmill #1 (during stance: − 0.4 ± 0.4%; during gait cycle: + 0.4 ± 0.5%), and the force-instrumented treadmill #2 (during stance: + 0.3 ± 0.2%; during gait cycle: + 0.4 ± 0.02%). Further, we identified a significant main effect of speed on the average TBV determined during the entire gait cycle (p = 0.013). Averaged over all treadmill types, the relative difference in average TBV determined during the gait cycle compared to the target speed was higher in the running compared to the walking speeds. However, there was a tendency that with faster running speeds these differences became lower, which was mostly affected by the behavior of the low-cost, non-instrumented treadmill (Fig. 4B). Overall, average TBV values ranged between 98 and 103% of the target belt speed. However, differences in average TBV between treadmills were apparent, particularly for the low-cost treadmill during the running trials (Fig. 4).

**Figure 5:**
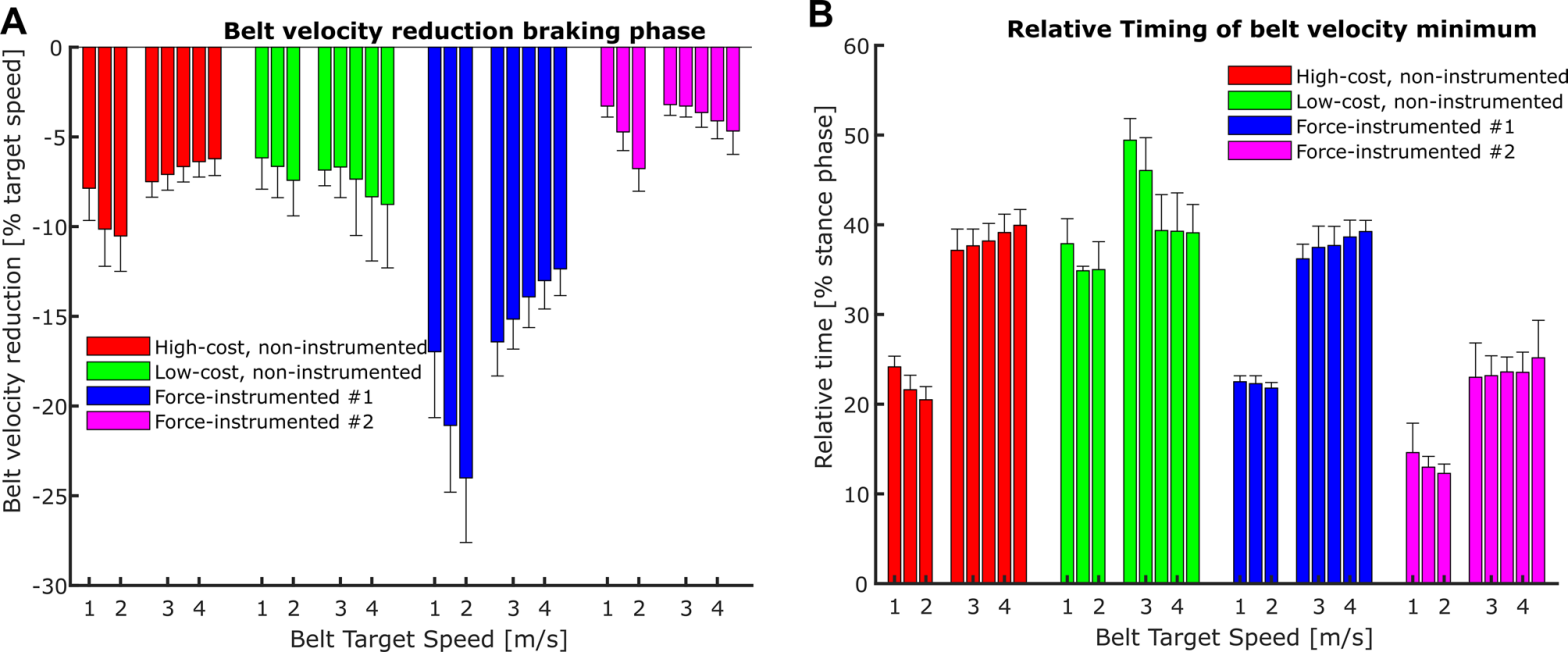
Treadmill belt velocity reduction (mean ± standard deviation) during the braking phase of stance (**A**) and its relative timing (**B**). We separated the bars of the walking speeds (1.0 m/s to 2.0 m/s) from the bars of the running speeds (2.5 m/s to 4.5 m/s).

**Figure 6:**
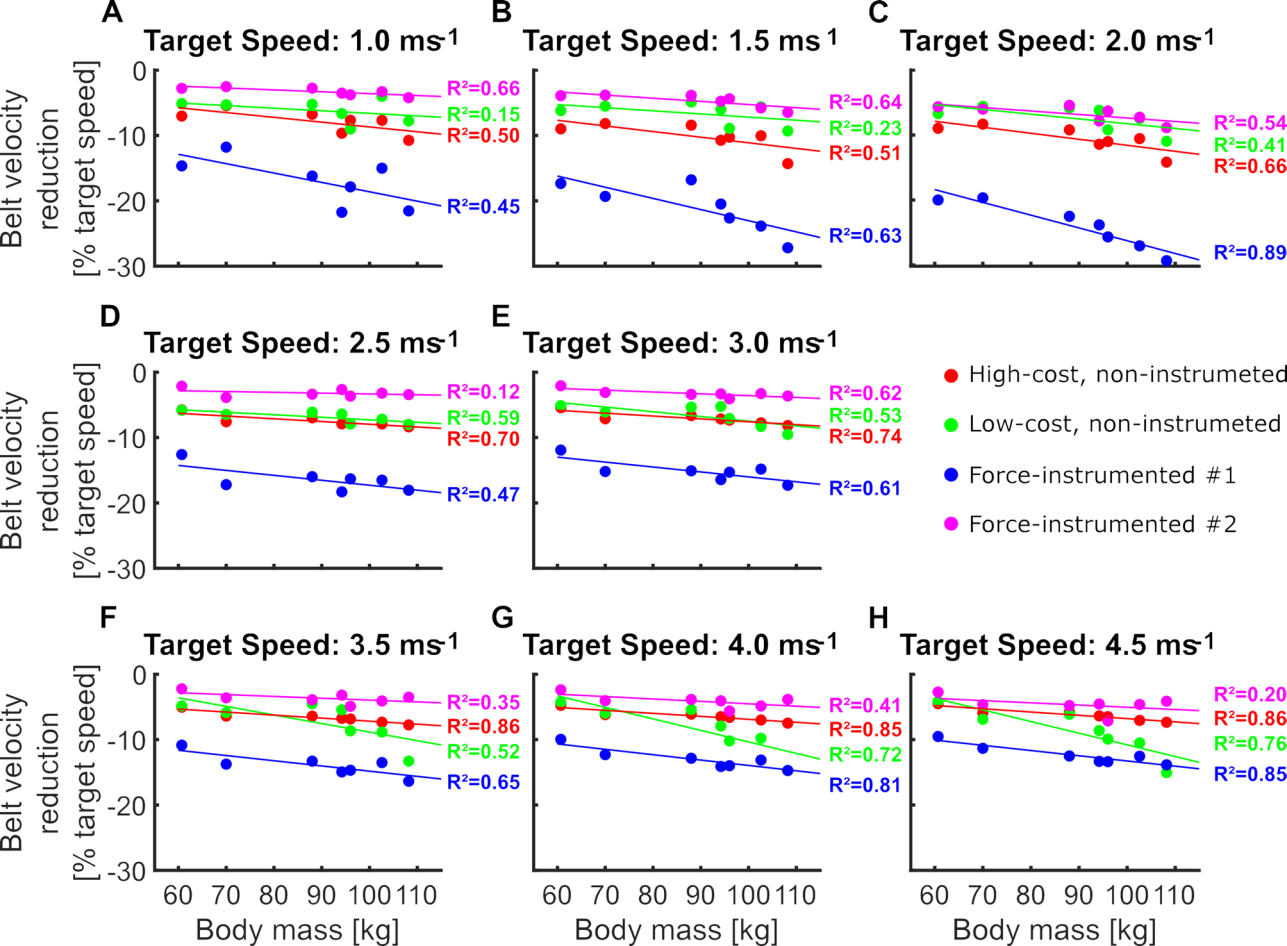
The relationship between the participants’ body mass and treadmill belt velocity reductions during the braking phase of stance at different walking (**A**–**C**) and running (**D**–**H**) speeds. Each dot represents the result of one particular participant, while colors differentiate between treadmill types. Colored lines were fitted using linear least squares estimation.

Furthermore, we found differences between treadmills in the TBV reductions and their timing in the early stance phase (Fig. 5A,B). Specificaly, we found a significant interaction effect of body mass, speed, and treadmill type (p < 0.001) on the TBV reduction during the braking phase of stance (Fig. 5A). This interaction effect highlights the different behaviors in response to variations in the participants mass and locomotor speed (Fig. 5A). Velocity reductions ranged between 2 and 10% for all treadmill types except for the instrumented treadmill #1, which showed reductions between 12 and 25% of the target speed (Fig. 5A). While higher body mass generally led to greater TBV reductions during the braking phase of stance, this relationship was specific for different treadmill types and locomotion speeds (Fig. 6). Averaging over all participants and locomotion speeds yielded the largest reductions in TBV during the braking phase for the instrumented treadmill #1 (− 16.6 ± 4.1%), followed by the high-cost, non-instrumented treadmill (− 7.8 ± 1.6%), the low-cost, non-instrumented treadmill (− 7.3 ± 0.9%), and the instrumented treadmill #2 (− 4.2 ± 1.2%).

When taking a closer look at the relative timing of minimal TBV reached during the braking phase of stance, we found significant main effects of speed (p < 0.001, Fig. 5B) and treadmill type (p < 0.001, Fig. 5B). The speed effect likely resulted from the fact that, averaged over all treadmills, at walking speeds the minimum TBV occurred relatively earlier (between 22.4 and 24.8% of stance) compared to running speeds (between 34.7 and 36.4%). Averaged over all speeds, minimum TBV during the braking phase occurred the earliest for the instrumented treadmill #2 (19.8 ± 5.5% of stance), followed by instrumented treadmill #1 (32.0 ± 8.1% of stance), the high-cost, non-instrumented treadmill (32.3 ± 8.6% of stance), and the low-cost, non-instrumented treadmill (40.1 ± 5.1% of stance). For almost all trials (30 of 32), minimum TBV was reached at or before 40% of the stance phase (Fig. 5B).

## Discussion

The first purpose of the present study was to develop an efficient algorithm that allows tracking of the instantaneous velocity of treadmill belts using standard motion capture technology without adding considerable time for post-processing.

Our method represents an extension to previously developed methods that either measured TBV fluctuation from single markers attached to the treadmill belt^1^ or which determined TBV during the stance phase from markers attached to the feet^16^. Compared to these previous approaches, our approach has several advantages. Since our method uses a higher number of markers to calculate TBV, it is inherently more robust and reliable. If, for example, a person walking or running on the treadmill is hiding a single marker attached to the treadmill belt, TBV cannot be determined accurately by methods relying on a single marker attached to the belt and must be interpolated during this period. This problem is aggravated when additional devices (e.g. oxygen consumption systems, additional weights, or exoskeletons) are carried by the subject moving on the treadmill. Furthermore, a single marker can track TBV only during the time when it is moving on the top of the treadmill, i.e. during 50% of the time.

When utilizing markers attached to the feet, TBV can only be determined during the stance phase and not during flight phases, which typically occur during running locomotion. Further, foot marker methods assume that during the entire stance phase there is no relative motion between foot and ground. However, this assumption not valid during most intervals of the stance phase, as illustrated by, e.g., the dorsiflexion and plantarflexion motion of the ankle joint to absorb or generate energy at the beginning or end of stance, respectively.

Another advantage of our method is the absence of additional post-processing work (e.g. labeling), making it very time efficient. We achieved this benefit by using “unlabeled markers”, i.e. markers that are not labeled automatically or manually through the motion capture software. “Unlabeled markers” are still given a kind of label (e.g. * plus a number in a Vicon system) by the motion capture system based on the timing of their first appearace in the file. However, further computations are needed to make use of the information stored in these marker coordinates. The results of our systematic analysis of TBVs revealed similar results for TBV fluctuation waveforms compared to previously reported results^1^, thus providing some evidence to support the validity of our method.

The second purpose of this study was to analyze the effects of treadmill type, locomotion speed, and body mass on TBV regulation. Significant main effects of treadmill type were identified in all parameters analyzed in this study. We further found significant main or interaction effects for locomotion speed and body mass for specific parameters of interest. Therefore, our hypothesis that TBV regulation would be affected by treadmill type, locomotion speed, and body mass was supported.

When looking at the average TBV determined during the stance phase or the entire gait cycle, it becomes apparent that relying on the displayed average speed when performing studies on treadmills is not advisable. We observed average differences of more than three percent between treadmill types for walking at a slow speed (Fig. 4). Therefore, one needs to be careful when comparing results for e.g., metabolic cost or locomotor economy, if they are collected from walking or running using different treadmill types. Essentially, the findings of this study suggest that it would be ideal to track TBV during every experiment or diagnostic analysis in which locomotion speed is used directly or for the calculation of derived variables (for e.g., running economy). If this approach is not feasible, it might be good practice to calibrate average TBV during every data collection and to occasionally verify that TBV fluctuations are small.

This suggestion is further emphasized by the finding of a significant main effect for speed on average TBV determined during the entire gait cycle. We found greater average TBV deviations at running compared to walking speeds. Therefore, even when using the same treadmill type, slightly different precision in the control of average locomotor speed can be expected, in particular when comparing walking with running gaits.

Furthermore, it is apparent from Fig. 3 that the patterns of TBV regulation vary between treadmill types in both their amplitude and timing. Therefore, it is likely that the way someone walks or runs on a treadmill is affected by the type of treadmill used. Hence, the comparability of treadmill to overground locomotion is also affected by the treadmill type used. This finding might partly explain the high heterogeneity of results that researchers have obtained when comparing overground to treadmill locomotion^11,17–19^. When trying to assess the validity of treadmill locomotion versus overground locomotion, specific criteria can be defined. For example, in running, the horizontal velocity of the center of mass changes as a result of the horizontal forces applied to the ground and the corresponding ground reaction force impulse. Previous research has established that when running at speeds between 3.0 and 5.0 m/s, the braking impulse of the ground reaction force is approximately equivalent to a 5% reduction in center of mass velocity and is generated from foot strike until just before 50% of stance^20^. When taking these values as a reference it appears that the TBV of 3 out of the 4 treadmill types in this study slows too much during initial stance compared to overground conditions, in particular the force-instrumented treadmill #1 (Fig. 5A). Further, when considering the timing of the peak minimal TBV, in all treadmill types tested the minimum TBV occurred too early (before approx. 50% of the stance phase (Fig. 5B)), compared to the data presented in a multitude of other studies^20,21^. Hence, for a better comparability to overground running conditions, treadmill manufacturers should try to modulate their TBV regulation towards meeting TBV profiles that are more consistent with typical overground conditions. At the same time, TBV regulation needs to be robust against varying ground force applications, e.g., due to differences in body mass and/or running speed between users.

Finally, it is important to note that the findings of this study have several limitations. Firstly, due to time restrictions, we could only analyze seven participants. We still found significant effects of the independent variables on TBV regulation. However, due to the low statistical power, we might have missed some effects. Further, while we attempted to collect data from different types of treadmills in new and worn-out conditions, our study does not provide a representative sample of the entire treadmill market. In particular, force-instrumented treadmill #1 showed the most pronounced fluctuations in TBV. Next to having a relatively worn out belt, it is not unlikely that these pronounced fluctuations were also the result of incorrect settings of the underlying TBV control algorithm of the treadmill. Therefore, future studies should explore other types of treadmills, and potentially also systematically modify factors like belt materials, the power supply of treadmill actuators, different states of lubrication or the effects of different running or walking styles on TBV regulation. We hope that our newly developed method will improve locomotion research and consequently lead to deeper insights in the future. We also did not compare our results of the average TBV to values obtained from unloaded treadmill belts at the respective target speeds. Such an analysis would provide greater insight to systematic errors of treadmill belt speed control independent of the characteristics of treadmill users or locomotion speed. Further, we did not directly assess whether the different motion capture systems used in different labs introduced any systematic or random errors to the assessment of TBV using our newly developed method. In each lab, we calibrated the motion capture systems following the guidelines provided by the manufacturers. Properly calibrated, modern 3D motion capture systems can capture marker data with high accuracy (< 0.1 mm error in position). Therefore, we believe that using different motion capture systems did not significantly affect our results. However, this statement needs to be verfified in future studies.

In conclusion, the results of this study highlight the need to monitor the velocity of the treadmill belt for locomotor research and the determination of applied diagnostics. We provide a novel method that is freely accessible and that allows tracking belt velocity in a time-efficient manner using standard motion capture systems. The code was developed using motion capture systems of three major manufacturers, and the authors hope to encourage the further adaptation of the code to data collected from other motion capture system manufacturers.

## Supplementary Information

Supplementary Information.

## References

[1] Savelberg HHCM, Vorstenbosch MATM, Kamman EH, van de Weijer JGW, Schambardt HC (1998). Intra-stride belt-speed variation affects treadmill locomotion. Gait Posture.

[2] Kram R, Taylor CR (1990). Energetics of running: A new perspective. Nature.

[3] Nilsson J, Thorstensson A (1989). Ground reaction forces at different speeds of human walking and running. Acta Physiol. Scand..

[4] Weyand PG, Sternlight DB, Bellizzi MJ, Wright S (2000). Faster top running speeds are achieved with greater ground forces not more rapid leg movements. J. Appl. Physiol..

[5] Schmidt-Nielsen K (1972). Locomotion: Energy cost of swimming, flying, and running. Science.

[6] Conley DL, Krahenbuhl GS (1980). Running economy and distance running performance of highly trained athletes. Med. Sci. Sports Exerc..

[7] Donelan JM, Kram R, Kuo AD (2002). Simultaneous positive and negative external mechanical work in human walking. J. Biomech..

[8] Zelik KE, Kuo AD (2010). Human walking isn’t all hard work: Evidence of soft tissue contributions to energy dissipation and return. J. Exp. Biol..

[9] Arampatzis A, Knicker A, Metzler V, Brüggemann G-P (2000). Mechanical power in running: A comparison of different approaches. J. Biomech..

[10] Zeni J, Richards J, Higginson JS (2008). Two simple methods for determining gait events during treadmill and overground walking using kinematic data. Gait Posture.

[11] Fellin RE, Manal K, Davis IS (2010). Comparison of lower extremity kinematic curves during overground and treadmill running. J. Appl. Biomech..

[12] Barre A, Armand S (2014). Biomechanical ToolKit: Open-source framework to visualize and process biomechanical data. Comput. Methods Programs Biomed..

[13] Mai P, Willwacher S (2019). Effects of low-pass filter combinations on lower extremity joint moments in distance running. J. Biomech..

[14] Willwacher S, Sanno M, Brüggemann G-P (2020). Fatigue matters: An intense 10 km run alters frontal and transverse plane joint kinematics in competitive and recreational adult runners. Gait Posture.

[15] Willwacher, S. *et al.* Surface stiffness and footwear affect the loading stimulus for lower extremity muscles when running. *J. Strength Cond. Res.*10.1519/JSC.0000000000003410 (2020). 10.1519/JSC.0000000000003410 32028460

[16] Fusco N, Crétual A (2008). Instantaneous treadmill speed determination using subject’s kinematic data. Gait Posture.

[17] Firminger CR (2018). Joint kinematics and ground reaction forces in overground versus treadmill graded running. Gait Posture.

[18] Miller JR (2019). A systematic review and meta-analysis of crossover studies comparing physiological, perceptual and performance measures between treadmill and overground running. Sports Med..

[19] Riley PO (2008). A kinematics and kinetic comparison of overground and treadmill running. Med. Sci. Sports Exerc..

[20] Munro CF, Miller DI, Fuglevand AJ (1987). Ground reaction forces in running: A reexamination. J. Biomech..

[21] Cavanagh PR, Lafortune MA (1980). Ground reaction forces in distance running. J. Biomech..

